# Evaluation of metformin performance on alloxan-induced diabetic rabbits

**DOI:** 10.25122/jml-2021-0417

**Published:** 2022-03

**Authors:** Youssef Shakuri Yasin, Wissam Sajid Hashim, Staar Mohammed Qader

**Affiliations:** 1. Nursing Department, Bilad Alrafidain University College, Diyala, Iraq; 2. Department of Physiology and Medical Physics, College of Medicine, University of Al-Muthanna, Samawa, Iraq; 3. Department of Microbiology, College of Medical Technology, Al-Kitab University, Kirkuk, Iraq

**Keywords:** metformin, alloxan, antidiabetic agent, antioxidant system, lipid profile

## Abstract

This study aimed to evaluate metformin as a widely used oral hypoglycemic agent and identify the effects on biochemical and antioxidant body systems of rabbits. Four groups of rabbits were randomly allocated as the control, the alloxan-induced diabetic, metformin-treated, and alloxan treated with metformin. The results revealed that alloxan leads to significant elevation in glucose (Glc) levels, malondialdehyde (MDA), low-density lipoprotein (LDL), very-low-density lipoprotein (VLDL), triglycerides (TGs), and total cholesterol (TCH), and a significant decline in high-density lipoprotein (HDL) and glutathione (GSH) as compared with the control group. Metformin alone caused a significant decline in Glc and HDL with significant elevation in LDL and MDA without significant changes in TCH, TGs, VLDL, and GSH. When metformin was offered as a treatment for alloxan-induced diabetic animals, it caused a significant decline in Glc, TCH, TGs, LDL, and VLDL levels with significant elevation in GSH and without a significant change in HDL and MDA. Metformin causes a decline in glucose levels due to its ability to decrease the use of substances hepatic cells use to create glucose and its ability to induce the enzymes participating in glucose oxidation.

## INTRODUCTION

The pathogenesis of both types of diabetes is considerably associated with radicals that cause lipids peroxidation [1]. *Diabetes mellitus* is caused by genetic and environmental alterations leading to pancreatic origin insulin or insulin insufficiency malfunctioning. The final upshot is a malfunction in the metabolism of carbohydrates, lipids, and proteins with severe alterations in redox reactions *vs.* systematic defensive antioxidants and other pathways [2]. Metformin is a well-known antidiabetic agent, especially for type II diabetes. It is known for activation of the pancreas and increasing insulin activity without increasing insulin secretion by beta cells. In addition, it causes a decreased appetite, so it is suitable for overweight patients, but it is also known to cause many gastrointestinal disturbances and even death [3].

## MATERIAL AND METHODS

Twenty four Albino rabbits with weights ranging from 1200 to 1400 grams and seven months of age were included in this study. The conditions of the experiment were all set in a very suitable manner. Alloxan was prepared according to the method used in a previous study [4]. The metformin solution was prepared every 48 hours to mantain its activity by dissolving 500 mg metformin tablets in 100 ml of distilled water and kept in the refrigerator [5].

The animals were allocated randomly into four groups of six rabbits:

1. Control group (G1): a dose of 5 ml distilled water administered orally;
2. The second group (G2): animals were injected subcutaneously with 150 mg/kg of alloxan one time to induce diabetes;
3. The third group (G3): animals were administered a daily dose of 5 ml metformin solution (25 mg/animal) for one month orally;
4. The fourth group (G4): animals were injected with alloxan in the same manner as the second group and then treated with metformin for one month. All the biochemical tests and histological examinations were accomplished.

### Statistical Analysis

The one-way ANOVA test was performed to identify the least significant difference (LSD) among the experimental groups. The Statistical Package for the Social Sciences (SPSS) program version 21 was used for data analysis.

## RESULTS

Alloxan caused a significant elevation in blood glucose (Glc), which significantly decreased when metformin was offered with alloxan without significance in the control group. Metformin alone caused a significant decline in glucose levels (Table 1).

**Table 1.** Glucose, Malondialdehyde, and Glutathione.

Groups	Glucose mg/100 ml	MDA µmol/L	GPx µmol/L
**G1**	94.67±1.93 ^b^	48.58±0.93 ^b^	4.93±0.034 ^ab^
**G2**	176±15.6 ^a^	81.9±7.63 ^a^	4.25±0.118 ^c^
**G3**	80.83±2.94 ^c^	71.43±5.35 ^a^	5.02±0.027 ^a^
**G4**	103.6±5.13 ^b^	74.75±6.58 ^a^	4.83±0.016 ^b^
**LSD**	13.84	22.85	0.19

The letters indicate the significance level. Data are summarized as means±standard deviation. G1 – Control; G2 – Alloxan; G3 – Metformin and G4 – Alloxan+Metformin.

Alloxan caused exacerbation in cholesterol concentrations (TCH) and triglycerides (TGs) while metformin alone or as a treatment to alloxan caused significant retardation compared to the alloxan treated group but not the control (Table 2). Alloxan caused a significant decline in HDL with exacerbation in LDL and very-low-density lipoprotein (VLDL), while metformin alone or as a treatment for alloxan caused more retardation in HDL *vs.* control or alloxan alone groups. Moreover, it caused a significant decline in LDL and VLDL compared with the alloxan group but not the control group. The antioxidant aspects were also affected with alloxan, causing a significant decline in glutathione (GSH) compared with all groups, while metformin alone did not affect the GSH either alone or as a treatment for alloxan (Table 1). All the treatment groups had a significant increase in MDA compared with the control group (P≤0.05) (Table 1).

**Table 2.** Lipids.

Groups	TC mg/100 ml	TG mg/100 ml	HDL mg/100 ml	LDL mg/100 ml	VLDL mg/100 ml
**G1**	94.67±1.93 ^c^	81.2±3.23 ^c^	24.93±0.03 ^a^	53.4±2.45 ^c^	19.43±0.567 ^c^
**G2**	140±15.6 ^a^	154.9±4.6 ^a^	18.25±0.11 ^b^	86.2±22.8 ^a^	32.83±7.46 ^a^
**G3**	87.34±2.94 ^c^	82.9±7.63 ^c^	15.02±0.02 ^c^	64.73±2.72 ^b^	19.7±0.434 ^c^
**G4**	103.6±5.13 ^b^	115.35±6.5 ^b^	17.83±0.01 ^bc^	67.63±2.28 ^b^	24.26±0.906 ^b^
**LSD**	8.93	32.45	6.68	11.33	4.65

The letters indicate the significance level. Data are summarized as means±standard deviation. G1 – Control; G2 – Alloxan; G3 – Metformin; G4 – Alloxan+Metformin.

## DISCUSSION

The elevated levels of glucose caused by alloxan are due to its action on pancreatic beta cells leading to obstruction of insulin production and then elevation of blood glucose [6]. Another confined mechanism is that alloxan affects the sulfhydryl groups of glucokinase, stopping their action in glycolysis and finally elevating blood glucose [7]. Metformin causes a decline in glucose levels, and this is due to its ability to decrease the utilization of substances used by hepatic cells to create glucose and to induce the enzymes participating in glucose oxidation [8]. Furthermore, it has a role in inhibiting enzymes responsible for glucose generation like phosphoenolpyruvate carboxykinase. It also causes an increase in the sensitivity of cellular receptors to insulin, causing an influx of glucose into cells and participating in inhibiting intestinal glucose absorption [9]. The elevated levels of cholesterol due to alloxan alone treatment result from induced diabetes, which causes increased intestinal cholesterol absorption in response to the activation of cholesterol acyltransferase [10]. When metformin was offered as a treatment with alloxan, it caused a decline in cholesterol because of the activation of ApoE mRNA by insulin [11]. The elevated triglycerides in the alloxan-treated group result from inhibiting lipoprotein lipase in fatty tissues because of insulin declination [12]. When metformin was introduced with alloxan, it caused a decline in triglycerides because of its action as an inhibitor of catecholamines, fatty tissues, and fat liberation. In addition, its activating role in insulin renders tissues more sensitive to insulin, and the activation of nitric oxide leads to a decrease in triglycerides levels [13]. HDL decline in alloxan-treated animals is preponderant because suppression of lipoprotein lipase is caused by alloxan and lipid peroxidation caused by the stress oxidation, which leads to degradation of tissue lipids and a decrease in HDL, which is responsible for cholesterol transport from tissues into the liver. The exacerbated activation of cholesterol ester transferase, which transfers cholesterol ester from HDL into VLDL, leaves the HDL molecules rich with TAGs [14]. The low densities of lipoproteins were significantly elevated before alloxan treatment, which might be due to elevations in chylomicrons or increased peroxidation of HDL [15, 16].

GSH was retarded before treatment with alloxan, which could be due to a decrease in the structural compounds essential for GSH formation like glutathione reductase or a decreased appetite predisposing animals to a decrease in nutritional antioxidants. Moreover, metformin caused a disturbance in GSH, relying upon a disturbance in redox reaction due to diabetes [17]. MDA increment was due to the increased ROS formation, which causes lipid peroxidation and destruction of cellular components [18].

## CONCLUSION

Metformin decreases blood glucose levels, but it also causes an elevation in oxidative stress status with fluctuations in the levels of lipoproteins, such as a decrease in HDL and elevation in LDL and VLDL. Therefore, metformin must be administered with monitoring and caution in patients with dyslipidemia.

## ACKNOWLEDGMENTS

### Conflict of interest

The authors declare no conflict of interest.

### Ethical approval

This study was approved by the Ethics Committee of the Scientific Research, College of Medicine, Al-Muthanna University, Iraq (approval no. 415/24.03.2022).

### Authorship

YSY contributed to conceptualizing the study, data collection and curation. WSH contributed to the methodology, data analysis, and editing. SMQ contributed to writing the original draft.
